# COVID-19, Renin-Angiotensin System, and Hematopoiesis

**DOI:** 10.4274/tjh.galenos.2020.2020.0174

**Published:** 2020-08-28

**Authors:** Rafiye Çiftçiler, İbrahim C. Haznedaroğlu

**Affiliations:** 1 Hacettepe University Faculty of Medicine, Department of Hematology, Ankara, Turkey

**Keywords:** COVID-19, Renin-angiotensin system, Hematopoiesis, SARS-CoV-2

## To the Editor,

We had initially indicated that there is a local hematopoietic bone marrow (BM) renin-angiotensin system (RAS), active in the physiological and pathological hematopoiesis (reviewed by Haznedaroglu  and Beyazit [1]). Turkish Journal of Hematology has already published two critical research papers on the impact of the local BM RAS on the pathobiological course of neoplastic hematological disorders [2,3].

Angiotensin-converting enzyme 2 (ACE2), an essential component of the RAS, is also the critical receptor of the SARS-CoV-2 virus, which is the responsible agent of the currently ongoing pandemic COVID-19. COVID-19 affects hematopoiesis [4] besides its well-known pulmonary involvement like other previous SARS viral infections [5]. The receptor binding domain for the spike protein of the SARS-CoV-2/ACE2 seems to be similar to that of the coronavirus strain involved in the 2002-2003 SARS outbreak [6].

Here we would like to point out that the hematopoietic effects of COVID-19/SARS viral infections including lymphopenia, leukoerythroblastosis, and macrophage activation syndrome may be linked to the viral effect on the local RAS [1] in the BM microenvironment. Leukoerythroblastic reactions associated with normocytic anemia, occasional nucleated red blood cells, mild anisocytosis, and rare dacrocytes were observed during the clinical course of COVID-19 infections [4]. Lymphopenia is a critical prognostic biomarker of the severity and hospitalization of the patients with  COVID-19 [7]. The imbalance between the ACE/angiotensin II/AT1R pathway and ACE2/angiotensin (1-7)/Mas receptor pathway in the RAS leads to the multi-system inflammation [8]. Likewise, macrophage activation syndrome is an essential integral part of the COVID-19 pathophysiology [9]. Similarly, enhanced expression of ACE in the lymphoma-associated macrophages in the lymph nodes in Hodgkin’s disease was previously demonstrated with regard to the local RAS [10].

The interrelationship between COVID-19, RAS, and hematopoiesis is not just an academic concern since the future modulation of the local RAS may be performed with directed RAS modulators, such as soluble ACE2, angiotensin (1-7), TXA127, and MAS receptor agonists. Topical soluble ACE2 has already been suggested as a ‘drug-of-hope’ for the pharmacobiological management of COVID-19 based on future controlled clinical trials [11].
